# Benefits of Puerarin on Metabolic Syndrome and Its Associated Cardiovascular Diseases in Rats Fed a High-Fat/High-Sucrose Diet

**DOI:** 10.3390/nu16091273

**Published:** 2024-04-25

**Authors:** Yu Mu, Yalin Yang, Shuang Jiang, Chilu Liu, Yanxing Han, Jiandong Jiang, Yuhong Wang

**Affiliations:** State Key Laboratory of Bioactive Substances and Function of Natural Medicine, Institute of Materia Medica, Chinese Academy of Medical Sciences and Peking Union Medical College, Beijing 100050, China

**Keywords:** metabolic syndrome, puerarin, cardiovascular disease, cardiac dysfunction, arterial stiffness, systemic inflammatory indices

## Abstract

Metabolic syndrome (MetS) is a cluster of risk factors for cardiovascular diseases (CVDs) that has become a global public health problem. Puerarin (PUE), the principal active compound of Pueraria lobata, has the effects of regulating glucose and lipid metabolism and protecting against cardiovascular damage. This study aimed to investigate whether dietary supplementation with PUE could ameliorate MetS and its associated cardiovascular damage. Rats were randomly divided into three groups: the normal diet group (NC), the high-fat/high-sucrose diet group (HFHS), and the HFHS plus PUE diet group (HFHS-PUE). The results showed that PUE-supplemented rats exhibited enhanced glucose tolerance, improved lipid parameters, and reduced blood pressure compared to those on the HFHS diet alone. Additionally, PUE reversed the HFHS-induced elevations in the atherogenic index (AI) and the activities of serum lactate dehydrogenase (LDH) and creatine kinase (CK). Ultrasonic evaluations indicated that PUE significantly ameliorated cardiac dysfunction and arterial stiffness. Histopathological assessments further confirmed that PUE significantly mitigated cardiac remodeling, arterial remodeling, and neuronal damage in the brain. Moreover, PUE lowered systemic inflammatory indices including C-reactive protein (CRP), neutrophil-to-lymphocyte ratio (NLR), monocyte-to-lymphocyte ratio (MLR), and systemic immune-inflammation index (SII). In conclusion, dietary supplementation with PUE effectively moderated metabolic disorders, attenuated systemic inflammation, and minimized cardiovascular damage in rats with MetS induced by an HFHS diet. These results provide novel insights into the potential benefits of dietary PUE supplementation for the prevention and management of MetS and its related CVDs.

## 1. Introduction

Metabolic syndrome (MetS) increases the risk of cardiovascular diseases (CVDs) and cardiovascular-related deaths. MetS is a cluster of metabolic abnormalities that are closely related to obesity. Defined by the American Heart Association (AHA) and the National Heart, Lung, and Blood Institute (NHLBI), MetS is diagnosed when at least three of the following five conditions coexist: abdominal obesity, elevated triglycerides (TG), reduced high-density lipoprotein cholesterol (HDL-C), hypertension, and impaired fasting glucose [1]. Global statistics show that approximately a quarter of the adult population is affected by MetS [2]. As social economy advances and lifestyles undergo transformation, the prevalence of MetS is increasing yearly, posing a significant global health problem. Interventions such as a Mediterranean diet, probiotics, statins, and anti-hyperglycemic agents have apparent benefits in the management of MetS [1]. However, nutraceuticals with minimal complications may be a promising field in the development of novel therapies.

Each component of MetS is regarded as an independent risk factor for CVDs, and the coexistence of these risk factors further increases the incidence of CVDs, including microvascular dysfunction, atherosclerosis, myocardial infarction, and heart failure (HF) [3]. There is an abundance of evidence demonstrating that patients with HF are burdened by several metabolic comorbidities included in MetS [3,4]. A study of a large cohort revealed that the presence of MetS was determined to be a significant predictor of HF [5]. In addition, accumulating evidence support the concept of arterial stiffness in MetS, potentially explaining the increased cardiovascular risk observed in individuals with MetS [6,7]. Multiple prospective studies involving patients with MetS showed that arterial stiffness is independently associated with increased cardiovascular morbidity and mortality [7,8,9,10]. As CVDs constitute the foremost cause of morbidity and mortality globally, it has become essential to investigate the preventive and therapeutic intervention for MetS and its associated CVDs to reduce the heavy burden of the disease.

Pueraria lobata, a medicinal and edible plant abundant in nutrients, has been receiving growing attention due to its remarkable pharmacological properties and health-promoting applications, as well as its advantages in terms of its low cost, low toxicity, and minimal side effects [11]. Puerarin (PUE), the principal active compound of the plant, exhibits a range of biological activities [11]. Numerous basic research and clinical trials have revealed that PUE plays a regulatory role in glucose and lipid metabolism and has the ability to mitigate oxidative stress and inflammation [12,13]. PUE is recognized as a safe and effective treatment for diabetes as well as CVDs such as coronary heart disease, arrhythmia, hypertension, and cerebral ischemia [14,15,16,17]. However, the efficacy of PUE in alleviating MetS and its related CVDs remains uncertain.

In the present study, we replicated a rat model mimicking human MetS with cardiovascular damage by using a long-term high-fat/high-sucrose (HFHS) diet. The aim was to investigate the effects of dietary PUE supplementation on diet-induced MetS and its associated cardiovascular damage. Here, we focused on assessing general metabolic parameters and cardiovascular injury associated with MetS, and also examined changes in the systemic inflammatory indices related to systemic chronic inflammation.

## 2. Materials and Methods

### 2.1. Animals and Diets

**Figure 1:**
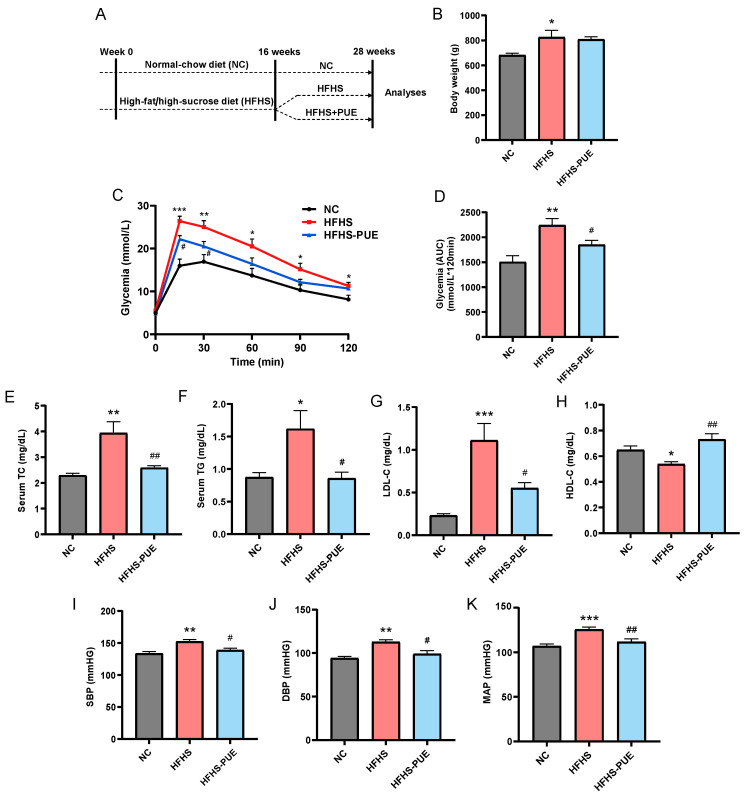
Effects of PUE on changes in metabolic parameters in HFHS diet rats. (**A**) Schematic diagram of experiments in rats; (**B**) body weight; (**C**) oral glucose tolerance test (OGTT); (**D**) OGTT area under the curve (AUC); serum levels of (**E**) total cholesterol (TC), (**F**) triglyceride (TG), (**G**) low-density lipoprotein cholesterol (LDL-C) and (**H**) high-density lipoprotein cholesterol (HDL-C); (**I**) systolic blood pressure (SBP); (**J**) diastolic blood pressure (DBP); (**K**) mean arterial pressure (MAP). NC: Normal-chow diet; HFHS: High-fat/high-sucrose diet; HFHS-PUE: High-fat/high-sucrose diet plus puerarin. Values were presented as mean ± SEM (n ≥ 5). * *p* < 0.05, ** *p* < 0.01, and *** *p* < 0.001 vs. NC; # *p* < 0.05 and ## *p* < 0.01 vs. HFHS.

Male Sprague Dawley rats (aged 8–10 weeks) were acquired from the Beijing Vital River Laboratory Animal Technology Co., Ltd. (Beijing, China). Rats were housed (2–3 rats per cage) with free access to standard food and water and maintained under controlled temperature (20–25 °C) and 12 h light/dark conditions for 1 week prior to experiments. All animal procedures were carried out in accordance with the guidelines and ethical principles of the Chinese Council on Animal Care. The protocol for all animal experiments was approved by the Animal Care and Use Committee of the Chinese Academy of Medical Sciences (No. 00003917). Animals were randomly divided into the following 3 groups: the NC group (n = 10), the HFHS group (n = 10), and the HFHS-PUE group (n = 10). The NC group had ad libitum access to a normal-chow diet, while the HFHS group and the HFHS-PUE group were subjected to experimental MetS through the high-fat/high-sucrose diet (fat 10%, sucrose 20%, cholesterol 2.5% and sodium cholate 0.5% by weight) from the Beijing Keao Xieli Feed Co., Ltd. (Beijing, China) for 28 weeks. During the last 12 weeks of the experiment, the HFHS-PUE group received an additional daily supplementation of 5% PUE (Aladdin, Shanghai, China). Figure 1A shows the schematic of the experimental protocol.

At the end of the experiment, body weight, glucose tolerance, lipid profiles, and blood pressure were assessed, respectively. Blood pressure was recorded using the tail cuff method by a CODA instrument (ADInstruments, Shanghai, China). An oral glucose tolerance test (OGTT) was conducted by giving the rats glucose orally at a dose of 2 g/kg body weight. The blood samples were then obtained from the tail vein at intervals of 0, 15, 30, 60, 90, and 120 min. Blood glucose levels were determined using the blood glucose meter (Yuwell, Danyang, China). The area under the curve (AUC) was calculated to assess glucose tolerance using GraphPad Prism 8.0 software.

Finally, animals were anesthetized with the inhalation of isoflurane (3–5%), and blood samples were collected from the abdominal aorta. The tissue of hearts, aortas, and brains were rapidly dissected out. Hearts were weighed and the heart index was determined by calculating the ratio of heart weight to body weight. Tissues were maintained in buffered 10% formalin for histopathological observation or kept at −80 °C for subsequent analysis.

### 2.2. Hemogram Analysis

Blood samples that were anticoagulated with ethylene diamine tetra acetic acid were assayed using the automated hematology analyzer CELL-DYN^®^ CD3700 (Abbott, Santa Clara, CA, USA). White blood cells count (WBCs) and neutrophils count (NEUs) were examined, and the neutrophil to lymphocyte ratio (NLR), monocyte to lymphocyte ratio (MLR), and systemic immune inflammation index (SII) were calculated using the following formulas [18]:

NLR = [absolute neutrophil count (ANC)]/[absolute lymphocyte count (ALC)]; MLR = ([absolute monocyte count (AMC)])/ALC; SII = [ANC × absolute platelet count (APC)]/ALC

### 2.3. Serum Biochemical Determination

The TG, total cholesterol (TC), low density lipoprotein cholesterol (LDL-C), HDL-C, activities of lactate dehydrogenase (LDH), and creatine kinase (CK) in the serum were measured by using commercial kits (BioSin Bio-Technology and Science Inc., Beijing, China). The serum C-reactive protein (CRP) was examined using a commercial kit (Elabscience, Wuhan, China).

### 2.4. Atherogenic Index (AI)

The AI was evaluated by the serum levels of TC and HDL-C. It was calculated according to the method of Hassan et al. [19], following the equation below:

AI = (TC − HDL-C)/(HDL-C)

### 2.5. Echocardiographic Analysis

At the end of the experiment, cardiac function was determined through standard transthoracic echocardiogram analysis using a Vevo2100 Imaging System (FUJIFILM VisualSonics, Toronto, ON, Canada). The left ventricular parameters including left ventricular anterior wall thickness (LVAW), left ventricular internal diameter (LVID), end-diastolic volume (EDV), ejection fraction (EF), and fractional shortening (FS) were measured via standard M-mode imaging. The mitral inflow E and A wave ratio (E/A) were evaluated using a pulse wave Doppler (PW Doppler) mode [20].

Aortic stiffness was assessed by measuring the pulse wave velocity (PWV) of abdominal aorta. Pulse waves were recorded from a PW Doppler image encompassing both the distal and proximal locations of the probes. The PWV was calculated using the following formula [21]:

PWV = (length of abdominal aorta)/(distal time delay − proximal time delay)

### 2.6. Histological Examination

Tissues were fixed in 10% buffered *formalin*. The samples were dehydrated in graded alcohol, embedded in paraffin wax, and cut into sections (4 μm thick) for hematoxylin and eosin (H&E) staining (left ventricle, abdominal aorta, and brain) and Masson trichrome staining (left ventricle and abdominal aorta). Changes in histopathology were observed under a microscope (Zeiss, Shanghai, China) and photographed. The images were quantitatively analyzed using the ImageJ software (Version 1.53). The cardiomyocyte cross-sectional area (CSA) based on HE staining was calculated to evaluate the cell sizes.

### 2.7. Immunofluorescence Analysis

The coronal brain slices of the right parietal cortex and hippocampus with a 20 μm thickness at intervals of 100 μm were sectioned. The selected sections were first fixed in ice-cold 4% paraformaldehyde for 15 min then washed 3 times with phosphate buffered saline (PBS). Brain sections were boiled in citric acid buffer for 5 min in a microwave oven and were treated with 0.3% Triton X100 and 10% goat serum for 1 h at room temperature, then incubated in primary antibodies for 2 h at room temperature and then overnight at 4 °C (1:300 rabbit anti-NeuN Polyclonal antibody, Proteintech, Wuhan, China). The sections were washed 3 times with PBS and then incubated in corresponding secondary antibodies in a dark room at room temperature for 2 h. Finally, all sections were counterstained and then stained with a dye containing 4′,6-diamidino-2-phenylindole (DAPI) and anti-fade reagent. Fluorescent images were observed under a microscope (Zeiss, Shanghai, China) and photographed. The NeuN-positive neurons were quantified using the ImageJ software (Version 1.53).

### 2.8. RNA Isolation and Reverse Transcription-Quantitative PCR (qRT-PCR)

Total RNA was extracted from the left ventricle with the PureLink™ RNA mini kit (Thermo Fisher Scientific, Waltham, MA, USA), followed by quantification with NanoDrop (Thermo Fisher Scientific, MA, USA). cDNA was synthesized using the HiFiScript cDNA Synthesis Kit (CoWin, Taizhou, China) and the qRT-PCR was carried out in the CFX Connect™ Real-Time PCR Detection System (Bio-Rad, Hercules, CA, USA) using the UltraSYBR Mixture (CoWin, Taizhou, China). The reference gene GAPDH was used for homogenization to correct and normalize the expression level of the target genes utilizing the 2^−ΔΔCT^ method. The following PCR primers sequences were used: Forward (F) and Reverse (R) for BNP: F: GAACAATCCACGATGCAGAAGC, R: GGGCCTTGGTCCTTTGAGAG; ANP, F: AGCCGAGACAGCAAACATCA, R: AGGTGGTCTAGCAGGTTCTTG; Collagen I, F: GGAGAGAGCATGACCGATGG, R: AAGTTCCGGTGTGACTCGTG; α-SMA, F: CATCCGACCTTGCTAACGGA, R: AATAGCCACGCTCAGTCAGG; GAPDH, F: TGATGGGTGTGAACCACGAG, R: GGCATGGACTGTGGTCATGA.

### 2.9. Statistical Analysis

All data are presented as mean ± SEM. The results were analyzed using GraphPad Prism 8.0. Depending on the design of the experiment, the data were analyzed using one-way analysis of variance (ANOVA) followed by Tukey’s post hoc test. A *p* value < 0.05 was considered statistically significant.

## 3. Results

### 3.1. PUE Improved Metabolic Parameters in HFHS Diet Rats

At the end of the experiment, we assessed the impact of PUE on the general symptoms of MetS, including glucose tolerance, lipid profiles, and blood pressure. The HFHS diet induced weight gain, glucose intolerance, lower level of HDL-C, and elevated levels of TC, TG, and LDL-C in rats (Figure 1B–H). Dietary PUE supplementation for 12 weeks significantly reversed these alterations induced by HFHS diet. PUE reduced the body weight of rats fed an HFHS diet, but the difference was not significant. Additionally, compared with the NC group, the SBP, DBP, and MAP were elevated in the HFHS group (*p* < 0.01, *p* < 0.01, and *p* < 0.001, respectively), and PUE supplementation significantly alleviated these changes (*p* < 0.05, *p* < 0.05, and *p* < 0.01, respectively; Figure 1I,J,K). These findings suggested that PUE protected against the general symptoms of MetS.

### 3.2. PUE Decreased the Cardiovascular Damage Indicators in HFHS Diet Rats

**Figure 2:**
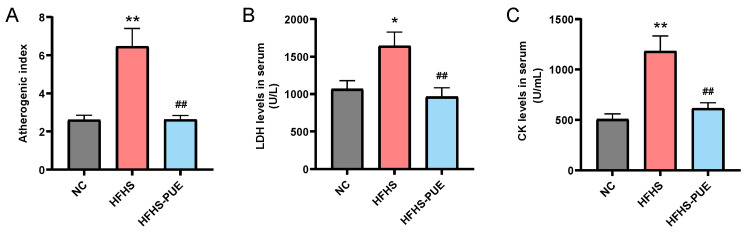
Effects of PUE on cardiovascular damage indicators. (**A**) Atherogenic index (AI); serum activities of (**B**) lactate dehydrogenase (LDH) and (**C**) creatine kinase (CK). NC: Normal-chow diet; HFHS: High-fat/high-sucrose diet; HFHS-PUE: High-fat/high-sucrose diet plus puerarin. Values were presented as mean ± SEM (n ≥ 5). * *p* < 0.05 and ** *p* < 0.01 vs. NC; ## *p* < 0.01 vs. HFHS.

AI, LDH, and CK serve as cardiovascular damage indicators. Compared with the NC group, the HFHS group exhibited a significant increase in AI (*p* < 0.01), accompanied by the elevated enzymatic activities of LDH (*p* < 0.05) and CK (*p* < 0.01). The administration of PUE effectively reversed these changes in HFHS diet rats (*p* < 0.01; Figure 2A–C). These findings suggested that PUE alleviated the cardiovascular damage associated with MetS induced by an HFHS diet.

### 3.3. PUE Mitigated Cardiac Dysfunction and Structural Remodeling in HFHS Diet Rats

**Figure 3:**
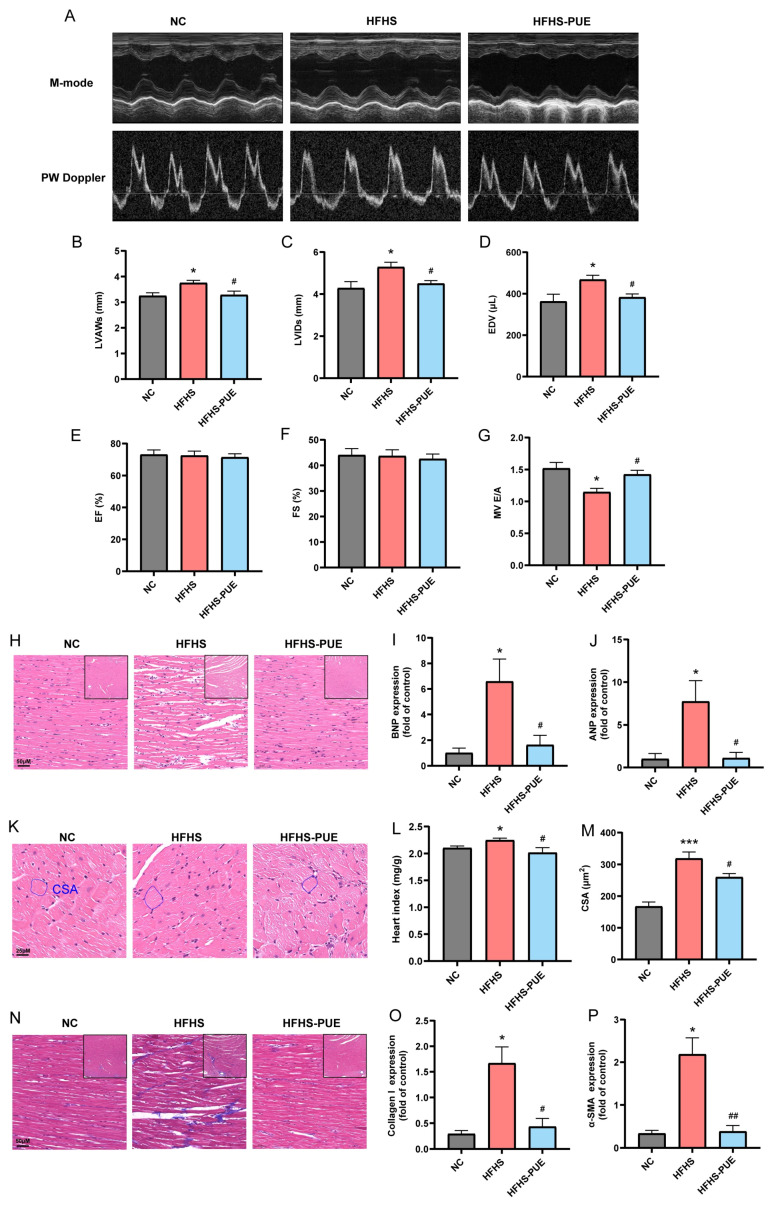
Effect of PUE on cardiac injury associated with MetS in HFHS diet rats. (**A**) Representative M-mode echocardiographic and PW Doppler images; (**B**) left ventricular anterior wall thickness (LVAW); (**C**) left ventricular internal diameter (LVID); (**D**) end-diastolic volume (EDV); (**E**) left ventricular ejection fraction (EF); (**F**) fractional shortening (FS); (**G**) the mitral inflow E and A wave ratio (E/A); (**H**) representative images of H&E-stained sections of the left ventricle (×20; framed zone: ×5); (**I**) BNP, (**J**) ANP mRNA expression in the left ventricle; (**K**) representative images of HE-stained sections of cross-sectional view of cardiomyocytes (×40); (**L**) heart index; (**M**) cross-sectional area of cardiomyocytes (CSA); (**N**) representative images of Masson-stained sections of the left ventricle (×20; framed zone: ×5); (**O**) Collagen I and (**P**) α-SMA mRNA expression in the left ventricle. NC: Normal-chow diet; HFHS: High-fat/high-sucrose diet; HFHS-PUE: High-fat/high-sucrose diet plus puerarin. Values were presented as mean ± SEM (n ≥ 5). * *p* < 0.05 and *** *p* < 0.001 vs. NC; # *p* < 0.05 and ## *p* < 0.01 vs. HFHS.

To evaluate the cardio-protective effect of PUE in MetS rats, transthoracic echocardiography was performed. Figure 3A shows representative echocardiography images indicating the systolic and diastolic function of heart. Rats fed an HFHS diet had thickened LVAW and increased LVID and EDV compared with the NC group (*p* < 0.05, Figure 3B–D). The effect of these processes was reversed by PUE supplementation (*p* < 0.05). An HFHS diet had no effect on the EF (Figure 3E) or the FS (Figure 3F) but decreased the E/A (*p* < 0.05; Figure 3G). However, PUE supplementation effectively reversed this reduction in the E/A (*p* < 0.05). Histopathological staining showed that PUE supplementation rescued broken myofilaments, corrected disordered sarcomeres, and reduced the percentage of interstitial fibrosis induced by an HFHS diet (Figure 3H,N). The heart index and CSA increased in the HFHS group, indicating cardiac hypertrophy (*p* < 0.05 and *p* < 0.001), which was reversed by PUE supplementation (*p* < 0.05, Figure 3K–M). qPCR results demonstrated that cardiac hypertrophy-related mRNAs, including brain natriuretic peptide (BNP) and atrial natriuretic peptide (ANP) (Figure 3I,J), and cardiac fibrosis-related mRNAs, including collagen I and α-smooth muscle actin (α-SMA) (Figure 3O,P), were upregulated in the HFHS group (*p* < 0.05) but downregulated after PUE supplementation (*p* < 0.05, *p* < 0.05, *p* < 0.05, and *p* < 0.01, respectively). These findings suggest that PUE has cardio-protective effects against cardiac dysfunction and structure remodeling associated with MetS.

### 3.4. PUE Ameliorated Arterial Stiffness in HFHS Diet Rats

**Figure 4:**
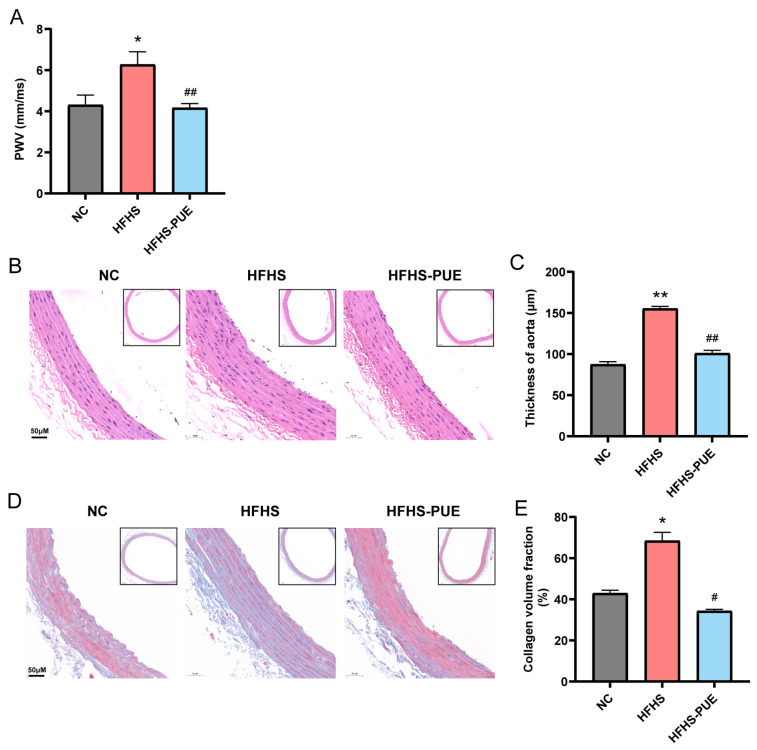
Effect of PUE on arterial stiffness associated with MetS in HFHS diet rats. (**A**) The pulse wave velocity (PWV) of abdominal aorta; (**B**) representative H&E-stained sections of the abdominal aorta (×20; framed zone: ×5); (**C**) measurement of the media thickness of the abdominal aorta; (**D**) representative Masson-stained sections of the abdominal aorta (×20; framed zone: ×5); (**E**) measurement of the collagen volume fraction of the abdominal aorta. NC: Normal-chow diet; HFHS: High-fat/high-sucrose diet; HFHS-PUE: High-fat/high-sucrose diet plus puerarin. Values were presented as mean ± SEM (A, n ≥ 5; D-E, n = 3). * *p* < 0.05 and ** *p* < 0.01 vs. NC; # *p* < 0.05 and ## *p* < 0.01 vs. HFHS.

To evaluate the effect of PUE on arterial stiffness, we utilized an ultrasound to measure the PWV of the abdominal aorta. The results showed that the PWV was accelerated in the HFHS group (*p* < 0.05) compared with the NC group, but significantly decelerated by PUE supplementation (*p* < 0.01; Figure 4A). H&E and Masson staining exhibited there was vascular remodeling in the abdominal aorta of the HFHS diet rats, characterized by disordered smooth muscle cells and interstitial fibrosis (Figure 4B,D). PUE supplementation reversed these changes, leading to a decrease in media thickness (*p* < 0.01) and the collagen volume fraction of the aorta (*p* < 0.05; Figure 4C,E). These findings suggest that PUE supplementation effectively protects against arterial stiffness related to MetS.

### 3.5. PUE Alleviated Neuronal Damage in HFHS Diet Rats

**Figure 5:**
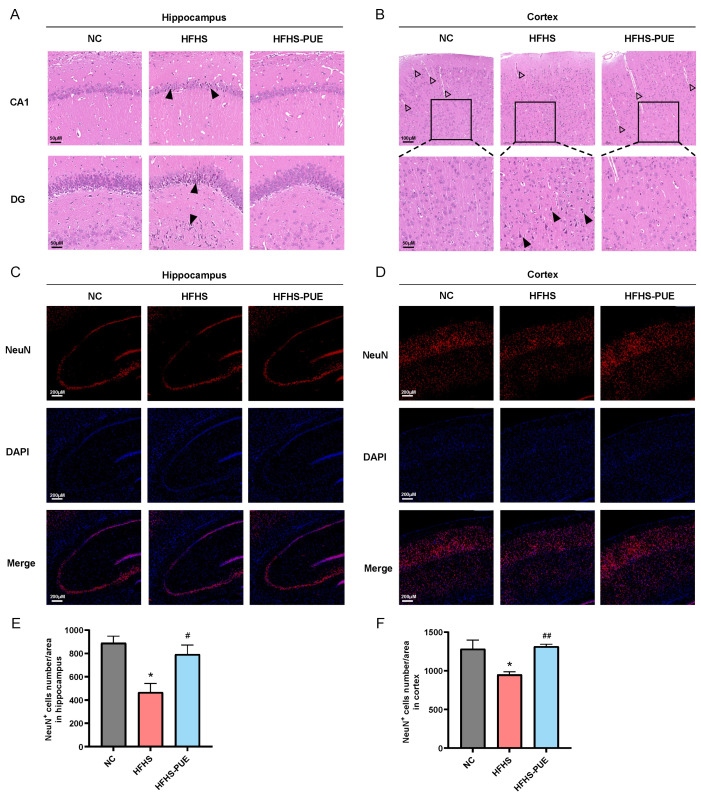
Effect of PUE on neuronal damage associated with MetS in HFHS diet rats. (**A**) Representative images of H&E staining of the CA1 and DG region of the hippocampus (×20); (**B**) representative images of H&E staining of the cortex (×10, ×20); (**C**) representative images of immunofluorescence staining of neurons in the hippocampus (×5) and (**D**) cortex (×5); (**E**) quantitative analysis of NeuN-positive cells number in the hippocampus and (**F**) cortex. Aberrant morphology of neurons (black arrow), capillaries (little arrow). NC: Normal-chow diet; HFHS: High-fat/high-sucrose diet; HFHS-PUE: High-fat/high-sucrose diet plus puerarin. Values were presented as mean ± SEM (n = 3). * *p* < 0.05 vs. NC; # *p* < 0.05 and ## *p* < 0.01 vs. HFHS.

The protective effect of PUE on neurons was evaluated by observing the histopathologic changes in the brain in HFHS diet rats. H&E staining results revealed that there were neuronal damages in the CA1 and DG regions of the hippocampus (Figure 5A) as well as in the cortex (Figure 5B) of HFHS diet rats, manifested as nuclear condensation and aberrant cell morphology. Furthermore, a reduction in the number of capillaries was observed in the cortex of HFHS diet rats. PUE supplementation significantly reversed these changes induced by an HFHS diet. Immunofluorescence staining of neuron labeling NeuN was further conducted, suggesting a significant reduction in neuron count in HFHS diet rats (Figure 5C–F). However, PUE supplementation significantly ameliorated this neuronal loss. These results indicated that PUE supplementation attenuated neuronal damage associated with MetS.

### 3.6. PUE Reduced the Systemic Inflammatory Indices in HFHS Diet Rats

**Figure 6:**
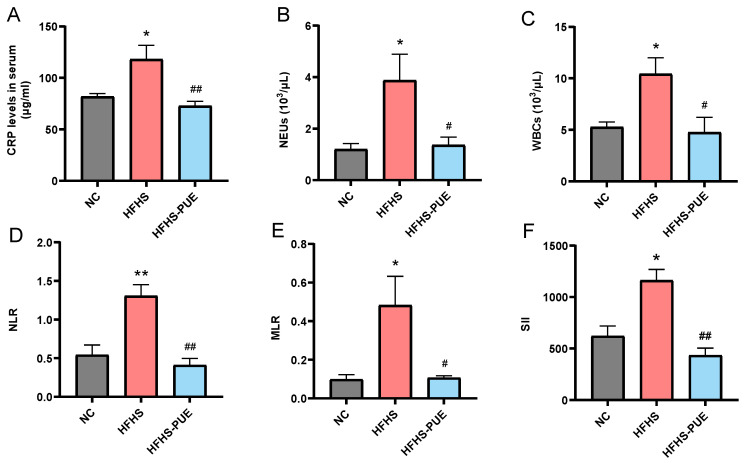
Effects of PUE on systemic inflammatory indices in HFHS diet rats. Comparative measurement of (**A**) the serum C-reactive protein (CRP) levels, (**B**) the neutrophils count (NEUs), (**C**) the white blood cells count (WBCs), (**D**) neutrophil-to-lymphocyte ratio (NLR), (**E**) monocyte to lymphocyte ratio (MLR), and (**F**) the systemic immune-inflammation index (SII). NC: Normal-chow diet; HFHS: High-fat/high-sucrose diet; HFHS-PUE: High-fat/high-sucrose diet plus puerarin. Values were presented as mean ± SEM (n ≥ 5). * *p* < 0.05 and ** *p* < 0.01 vs. NC; # *p* < 0.05 and ## *p* < 0.01 vs. HFHS.

MetS has been shown to be associated with low-grade systemic inflammation. The serum level of CRP was increased in the HFHS group (*p* < 0.05) but significantly decreased by PUE supplementation (*p* < 0.01; Figure 6A). Hemogram analysis indicated that NEUs and WBCs increased in the HFHS group (*p* < 0.05) and significantly decreased in the HFHS-PUE group (*p* < 0.05; Figure 6B,C). NLR, MLR, and SII, as new inflammatory markers, have the potential to predict the clinical outcome of cardiovascular and cerebrovascular diseases [18]. Compared with the NC group, NLR, MLR, and SII significantly increased in the HFHS group (*p* < 0.01, *p* < 0.05, and *p* < 0.05, respectively), and were significantly decreased by PUE supplementation (*p* < 0.01, *p* < 0.05, and *p* < 0.01, respectively; Figure 6 D–F). These results indicated that PUE may play a protective role on the heart, brain, and vascular in MetS, reducing the risk of CVDs and improving the prognosis of the disease by reducing systemic inflammation.

## 4. Discussion

In recent years, numerous studies have demonstrated the beneficial effects of PUE on the regulation of glucose and lipid metabolism [12,13]. A review indicates that PUE is effective in mitigating the initiation and progression of various diseases, including obesity, diabetes, hypertension, atherosclerosis, cardiac ischemia, cardiac arrhythmia, cardiac hypertrophy, ischemic stroke, and cognition decline, by reducing oxidative stress and inflammation [12]. In this study, for the first time, we systematically examined the impact of PUE on MetS and its associated CVDs in rats fed an HFHS diet.

We replicated a rat model of human MetS with cardiovascular damage by subjecting animals to a long-term HFHS diet for 28 weeks. The HFHS diet induced signs of MetS with cardiovascular injury, such as weight gain, impaired glucose tolerance, elevated blood pressure, abnormal lipid profiles, cardiac diastolic dysfunction, arterial stiffness, and neuronal damage in rats. However, dietary supplementation with PUE ameliorated these alterations induced by the HFHS diet. A review has summarized the mechanisms by which PUE regulates glucose and lipid metabolism both in vivo and vitro [13]. These mechanisms include inhibiting the release of glucose and free fatty acid (FFA), modulating the transport of glucose and fatty acid (FA), reducing the synthesis of glucose and FA, promoting β-oxidation, enhancing insulin secretion and sensitivity, and alleviating oxidative stress and inflammatory responses. A clinical trial involving 18–50-year-old men without a history of CVDs indicated that PUE supplementation had lower fasting glucose compared to the placebo group [22]. Furthermore, the accumulating evidence indicate that PUE has anti-hypertensive effect in various animal models of hypertension, including spontaneously hypertensive rats (SHR), Ang II-infused hypertensive rats, and renovascular hypertensive rats [23]. In this study, we have shown for the first time that PUE reduces blood pressure in rats with MetS-associated hypertension. Additionally, it is reported that PUE ameliorates metabolic-dysfunction-associated fatty liver disease [24,25]. Our recent findings were consistent with previous studies that PUE improved the serum aspartate transaminase (AST) and alanine aminotransferase (ALT) level, reduced liver fat accumulation, and exhibited the protective effect on MetS-associated liver dysfunction. PUE supplementation reduced the body weight in rats fed an HFHS diet, but the difference was not significant. This is similar to a previous report [26]. Collectively, this is the first in vivo study to evaluate whether dietary supplementation with PUE could ameliorate various manifestations of MetS in rats fed an HFHS diet.

High levels of TC, TG, and LDL-C, lower HDL-C, and an increased AI are all indicators of atherogenic dyslipidemia [27]. It is reported that PUE mitigated the hypercholesterolemic diet-induced elevation of TC in both serum and liver, leading to a significant decrease in the AI [28]. Clinical studies have shown that the AI was significantly higher in individuals with MetS [29]. In our study, rats fed an HFHS diet exhibited a significantly increased AI, but PUE supplementation reversed this change. The result suggested that PUE might reduce the risk of MetS-associated CVDs.

The protective role of PUE on cardiac function have been demonstrated in numerous studies. In a rat model of coronary artery disease, PUE decreased circulating markers of cardiac damage (CK, CK-MB, LDH, troponin) and protected against cardiomyocyte damage [30]. In mice with myocardial fibrosis, PUE decreased fibrosis by inhibiting NF-kB activation and collagen deposition, thereby enhancing cardiac performance [31]. PUE has also been shown to alleviate CVDs by reducing infarct size, inflammatory markers, and FFA levels in those with myocardial infarction [32,33]. MetS components can individually serve as independent risk factors for HF development, while there is also a high prevalence of MetS among HF patients [3]. Overnutrition is the primary trigger leading to insulin resistance, neurohormonal activation, microvascular circulation impairment, oxidative stress and inflammation. These conditions ultimately result in myocardial cell death, extracellular fibrosis, and altered myocardial-endothelial interactions [34]. In our study, an HFHS diet caused cardiac diastolic dysfunction and cardiac structure remodeling with broken myofilaments, disordered sarcomeres, and interstitial fibrosis in rats. However, EF and FS remained within normal ranges. These findings are consistent with the features of HF with preserved EF (HFpEF), a prevalent type of HF characterized by normal systolic function but diastolic dysfunction of heart [35]. HFpEF has been considered as the predominant type of HF worldwide and its incidence is escalating yearly with an annual mortality rate of approximately 15% [36,37]. Mechanistically, it has been postulated that chronic low-grade systemic inflammation linked to MetS contributes to ventricular remodeling in HFpEF [35]. The enzymatic activities of LDH and CK were significantly increased in the HFHS diet rats. PUE supplementation reduced the LDH and CK activities, improved cardiac diastolic dysfunction, and reversed the cardiac histopathological alternations induced by HFHS diet. PUE also decreased the expression of cardiac hypertrophy and fibrosis-related mRNAs. These findings suggested that PUE could protect against the HFpEF associated with MetS.

Hypertension, diabetes, dyslipidemia, and obesity all contributed to arterial stiffness. Accordingly, MetS is closely related to arterial stiffness, which is characterized by excessive fibrosis and loss of arterial elasticity that result in a diminished arterial storage capacity and an accelerated PWV along the vessel wall [21,38,39,40,41]. Arterial stiffness leads to an increase in SBP, promotes left ventricular hypertrophy, and might progress to HF [6]. Hence, MetS associated with arterial stiffness has been established as one of the major risk factors for the progression of CVDs [42]. There are limited evidence demonstrating the beneficial effect of PUE on arterial stiffness. It was reported that the administration of PUE resulted in a mild reduction in carotid artery thickness and the inner diameter in SHR [43]. Additionally, PUE has exhibited the ability to inhibit vascular calcification in vivo [44] and in vitro [45]. A randomized controlled trial suggested that 24 weeks of intravenous treatment with PUE reduced carotid intima-media thickness in subjects with rheumatoid arthritis [46]. Our findings exhibited that prolonged consumption of an HFHS diet leads to accelerated PWV and structural abnormalities in the aorta, including increased thickness and fibrosis. However, dietary supplementation with PUE reduced the thickness of aorta, decreased fibrosis, and restored the PWV. Collectively, our results firstly reported that dietary supplementation with PUE has the potential to reverse arterial stiffness induced by MetS and restore vascular compliance. It is important to note that arterial stiffness tends to worsen as the number of MetS components increases [47,48,49]. Conversely, recovery from MetS has been shown to alleviate arterial stiffness [47]. Therefore, further research is warranted to determine whether PUE directly contributes to the mitigation of arterial stiffness or indirectly through alleviating symptoms of MetS components.

Patients with MetS have an increased risk of developing cognitive impairment [50]. Hypercholesterolemia triggered by diet exacerbates neuroinflammatory response, provokes atherosclerosis, and increases brain blood barrier permeability, resulting in neuronal damage and cognitive impairment [51,52,53]. Arterial stiffness not only promotes left ventricular hypertrophy but also imposes a significant hemodynamic burden on cerebral circulation, leading to microvascular damage [54]. Additionally, HF leads to cerebral hypoperfusion and chronic cerebral hypoxia, ultimately resulting in cerebral microbleeds, neurodegeneration, and cognitive impairment [55]. It is reported that PUE can protect against neuronal injury induced by different etiologies, such as aging and hypertension [12]. In our study, rats with diet-induced MetS exhibited neuronal damage and loss, and a reduction in cortical and hippocampal capillaries. However, these alterations were reversed through PUE supplementation. These findings do not directly indicate that rats fed an HFHS diet have cognitive impairment. However, structural changes and a reduction in the number of neurons may affect the speed at which information is transmitted and processed between neurons, thus affecting cognitive function [56,57]. Therefore, our findings suggest that PUE may serve as a preventive measure against neurological impairment associated with MetS and reduce the risk of cognitive impairment.

Markers of systemic inflammation, including CRP, interleukin 6 (IL-6), and tumor necrosis factor-alpha (TNF-α) have been found to be elevated in patients with MetS [2]. Recent evidence suggests that oxidative stress and chronic low-grade systemic inflammation may underlie the pathophysiological process of MetS and CVDs [58]. PUE exerts beneficial effects by alleviating oxidative stress and adverse inflammatory events in the heart, arteries, and brain, thereby impeding the pathogenesis of obesity, diabetes, hypertension, atherosclerosis, cardiac dysfunction, and cognitive decline [12]. Recently, the systemic inflammatory indices, including CRP, NLR, MLR, and SII, serve as indicators of systemic inflammation and prognostic marker for chronic CVDs [59,60,61,62]. Our findings revealed elevated levels of them in rats with MetS. However, dietary supplementation with PUE effectively decreased these inflammatory indices. These results indicate that alleviating systemic inflammation may be the potential mechanism by which PUE exerts its protective effect. In the future, we will study the precise molecular mechanisms by which PUE regulates systemic inflammation at the cellular level.

Despite the promising results, there are several limitations in this study. Firstly, the study duration was relatively short, and it is imperative to conduct longer-term studies in order to evaluate the sustained effects of PUE supplementation. Secondly, the precise mechanisms responsible for the benefit of PUE still need to be fully elucidated. Finally, the utilization of a rat model may not comprehensively predict the intricate physiological reactions in humans. In the future, it would be valuable to investigate the long-term effects of PUE supplementation on lifespan and overall health in animal models. Further studies are required to elucidate the precise mechanisms through which PUE exerts its beneficial effects. Additionally, clinical trials are warranted to assess the efficacy and safety of PUE supplementation in individuals with MetS and related CVDs. Such studies would provide valuable insights into the potential of PUE as a supplement agent for the prevention and management of these conditions.

## 5. Conclusions

In conclusion, our study showed that rats fed an HFHS diet exhibited the general manifestations of MetS and its associated cardiovascular injury. Dietary supplementation with PUE was found to improve glucose intolerance and blood lipid parameters, lower blood pressure, improve cardiac diastolic dysfunction and cardiac structural remodeling, alleviate arterial stiffness, regulate the body’s inflammatory state, and reduce the risk of cognitive impairment. These results showed that the effects of PUE on MetS and its associated CVDs might be multifaceted. However, further research is necessary to confirm these findings in humans and elucidate the underlying mechanisms responsible for the beneficial effects of PUE.

## Data Availability

The data presented in this study are available on request from the corresponding author due to privacy reasons.

## References

[1] Fahed G., Aoun L., Bou Zerdan M., Allam S., Bou Zerdan M., Bouferraa Y., Assi H.I. (2022). Metabolic Syndrome: Updates on Patho-physiology and Management in 2021. Int. J. Mol. Sci..

[2] Gupta A., Gupta V. (2010). Metabolic syndrome: What are the risks for humans?. Biosci. Trends.

[3] Purwowiyoto S.L., Prawara A.S. (2021). Metabolic syndrome and heart failure: Mechanism and management. Med. Pharm. Rep..

[4] Arcopinto M., Schiavo A., Salzano A., Bossone E., D’assante R., Marsico F., Demelo-Rodriguez P., Baliga R.R., Cittadini A., Marra A.M. (2019). Metabolic Syndrome in Heart Failure: Friend or Foe?. Heart Fail. Clin..

[5] Ingelsson E., Arnlöv J., Lind L., Sundström J. (2006). Metabolic syndrome and risk for heart failure in middle-aged men. Heart.

[6] Stehouwer C.D., Henry R.M., Ferreira I. (2008). Arterial stiffness in diabetes and the metabolic syndrome: A pathway to cardiovascular disease. Diabetologia.

[7] Laurent S., Boutouyrie P., Asmar R., Gautier I., Laloux B., Guize L., Ducimetiere P., Benetos A. (2001). Aortic stiffness is an independent predictor of all-cause and cardiovascular mortality in hypertensive patients. Hypertension.

[8] Cruickshank K., Riste L., Anderson S.G., Wright J.S., Dunn G., Gosling R.G. (2002). Aortic pulse-wave velocity and its relationship to mortality in diabetes and glucose intolerance: An integrated index of vascular function?. Circulation.

[9] Willum-Hansen T., Staessen J.A., Torp-Pedersen C., Rasmussen S., Thijs L., Ibsen H., Jeppesen J. (2006). Prognostic value of aortic pulse wave velocity as index of arterial stiffness in the general population. Circulation.

[10] Mattace-Raso F.U., Van Der Cammen T.J., Hofman A., Van Popele N.M., Bos M.L., Schalekamp M.A., Asmar R., Reneman R.S., Hoeks A.P., Breteler M.M. (2006). Arterial stiffness and risk of coronary heart disease and stroke: The Rotterdam Study. Circulation.

[11] Wang D., Bu T., Li Y., He Y., Yang F., Zou L. (2022). Pharmacological Activity, Pharmacokinetics, and Clinical Research Progress of Puerarin. Antioxidants.

[12] Zhang L., Liu L., Wang M. (2021). Effects of puerarin on chronic inflammation: Focus on the heart, brain, and arteries. Aging Med..

[13] Jing X., Zhou J., Zhang N., Zhao L., Wang S., Zhang L., Zhou F. (2022). A Review of the Effects of Puerarin on Glucose and Lipid Metabolism in Metabolic Syndrome: Mechanisms and Opportunities. Foods.

[14] Liu X., Huang R., Wan J. (2023). Puerarin: A potential natural neuroprotective agent for neurological disorders. Biomed. Pharmacother..

[15] Zhu T., Wang L., Wang L.P., Wan Q. (2022). Therapeutic targets of neuroprotection and neurorestoration in ischemic stroke: Applications for natural compounds from medicinal herbs. Biomed. Pharmacother..

[16] Xu H., Yu S., Lin C., Dong D., Xiao J., Ye Y., Wang M. (2024). Roles of flavonoids in ischemic heart disease: Cardioprotective effects and mechanisms against myocardial ischemia and reperfusion injury. Phytomedicine.

[17] Qin W., Guo J., Gou W., Wu S., Guo N., Zhao Y., Hou W. (2022). Molecular mechanisms of isoflavone puerarin against cardiovascular diseases: What we know and where we go. Chin. Herb. Med..

[18] Zhao Y., Shao W., Zhu Q., Zhang R., Sun T., Wang B., Hu X. (2023). Association between systemic immune-inflammation index and metabolic syndrome and its components: Results from the National Health and Nutrition Examination Survey 2011–2016. J. Transl. Med..

[19] Hassan S., El-Twab S.A., Hetta M., Mahmoud B. (2011). Improvement of lipid profile and antioxidant of hypercholesterolemic albino rats by polysaccharides extracted from the green alga Ulva lactuca Linnaeus. Saudi J. Biol. Sci..

[20] Nagueh S.F., Smiseth O.A., Appleton C.P., Byrd B.F., Dokainish H., Edvardsen T., Flachskampf F.A., Gillebert T.C., Klein A.L., Lancellotti P. (2016). Recommendations for the Evaluation of Left Ventricular Diastolic Function by Echocardiography: An Update from the American Society of Echocardiography and the European Association of Cardiovascular Imaging. J. Am. Soc. Echocardiogr..

[21] Cecelja M., Chowienczyk P. (2009). Dissociation of aortic pulse wave velocity with risk factors for cardiovascular disease other than hypertension: A systematic review. Hypertension.

[22] Kwok M.K., Leung G.M., Xu L., Tse H.F., Lam T.H., Schooling C.M. (2022). Effect of puerarin supplementation on cardiovascular disease risk factors: A randomized, double-blind, placebo-controlled, 2-way crossover trial. Biomed. Pharmacother..

[23] Zhou Y.X., Zhang H., Peng C. (2021). Effects of Puerarin on the Prevention and Treatment of Cardiovascular Diseases. Front. Pharmacol..

[24] Yang M., Xia L., Song J., Hu H., Zang N., Yang J., Zou Y., Wang L., Zheng X., He Q. (2023). Puerarin ameliorates metabolic dysfunction-associated fatty liver disease by inhibiting ferroptosis and inflammation. Lipids Health Dis..

[25] Wang S., Yang F.J., Shang L.C., Zhang Y.H., Zhou Y., Shi X.L. (2019). Puerarin protects against high-fat high-sucrose diet-induced non-alcoholic fatty liver disease by modulating PARP-1/PI3K/AKT signaling pathway and facilitating mitochondrial homeostasis. Phytother. Res..

[26] Noh J.W., Yang H.K., Jun M.S., Lee B.C. (2022). Puerarin Attenuates Obesity-Induced Inflammation and Dyslipidemia by Regulating Macrophages and TNF-Alpha in Obese Mice. Biomedicines.

[27] Barua L., Faruque M., Banik P.C., Ali L. (2019). Atherogenic index of plasma and its association with cardiovascular disease risk factors among postmenopausal rural women of Bangladesh. Indian. Heart J..

[28] Yan L.P., Chan S.W., Chan A.S., Chen S.L., Ma X.J., Xu H.X. (2006). Puerarin decreases serum total cholesterol and enhances thoracic aorta endothelial nitric oxide synthase expression in diet-induced hypercholesterolemic rats. Life Sci..

[29] Sabarinathan M., Ds D.R., Ananthi N., Krishnan M. (2022). Atherogenic index of plasma, lipid accumulation and visceral adiposity in metabolic syndrome patients. Bioinformation.

[30] Zhao L., Wang L., Zhang D., Chen Y., Jin F. (2021). Puerarin alleviates coronary heart disease via suppressing inflammation in a rat model. Gene.

[31] Chen R., Xue J., Xie M. (2012). Puerarin prevents isoprenaline-induced myocardial fibrosis in mice by reduction of myocardial TGF-β1 expression. J. Nutr. Biochem..

[32] Xiao L.Z., Huang Z., Ma S.C., Zen Z., Luo B., Lin X., Xu X. (2004). [Study on the effect and mechanism of puerarin on the size of infarction in patients with acute myocardial infarction]. Zhongguo Zhong Xi Yi Jie He Za Zhi.

[33] Xiao L.Z., Gao L.J., Ma S.C. (2005). [Comparative study on effects of puerarin and granulocyte colony-stimulating factor in treating acute myocardial infarction]. Zhongguo Zhong Xi Yi Jie He Za Zhi.

[34] Gargiulo P., Marsico F., Renga F., Dell’aversana S., Esposito I., Marciano C., Dellegrottaglie S., Perrone-Filardi P., Paolillo S. (2020). The metabolic syndrome in heart failure: Insights to specific mechanisms. Heart Fail. Rev..

[35] Capone F., Sotomayor-Flores C., Bode D., Wang R., Rodolico D., Strocchi S., Schiattarella G.G. (2023). Cardiac metabolism in HFpEF: From fuel to signalling. Cardiovasc. Res..

[36] Redfield M.M., Borlaug B.A. (2023). Heart Failure with Preserved Ejection Fraction: A Review. JAMA.

[37] Borlaug B.A. (2020). Evaluation and management of heart failure with preserved ejection fraction. Nat. Rev. Cardiol..

[38] Li Z., Froehlich J., Galis Z.S., Lakatta E.G. (1999). Increased expression of matrix metalloproteinase-2 in the thickened intima of aged rats. Hypertension.

[39] Wilkinson I.B., Prasad K., Hall I.R., Thomas A., Maccallum H., Webb D.J., Frenneaux M.P., Cockcroft J.R. (2002). Increased central pulse pressure and augmentation index in subjects with hypercholesterolemia. J. Am. Coll. Cardiol..

[40] Mitchell G.F., Guo C.Y., Benjamin E.J., Larson M.G., Keyes M.J., Vita J.A., Vasan R.S., Levy D. (2007). Cross-sectional correlates of increased aortic stiffness in the community: The Framingham Heart Study. Circulation.

[41] Uchida J., Machida Y., Iwai T., Kuwabara N., Kabei K., Naganuma T., Kumada N., Nakatani T. (2013). Glucose intolerance is associated with increased intimal-medial thickness of the carotid artery and increased pulse-wave velocity in renal transplant recipients. Transplant. Proc..

[42] Chirinos J.A., Segers P., Hughes T., Townsend R. (2019). Large-Artery Stiffness in Health and Disease: JACC State-of-the-Art Review. J. Am. Coll. Cardiol..

[43] Fang X., Dong S., Wu Y., He Y., Lu M., Shi D., Feng N., Yin S., Jiang Y., Zhang A. (2021). Ameliorated biomechanical properties of carotid arteries by puerarin in spontaneously hypertensive rats. BMC Complement. Med. Ther..

[44] Liu H., Zhang X., Zhong X., Li Z., Cai S., Yang P., Ou C., Chen M. (2019). Puerarin inhibits vascular calcification of uremic rats. Eur. J. Pharmacol..

[45] Lu Q., Xiang D.X., Yuan H.Y., Xiao Y., Yuan L.Q., Li H.B. (2014). Puerarin attenuates calcification of vascular smooth muscle cells. Am. J. Chin. Med..

[46] Yang M., Luo Y., Liu T., Zhong X., Yan J., Huang Q., Tao J., He Q., Guo M., Hu Y. (2018). The Effect of Puerarin on Carotid Intima-media Thickness in Patients with Active Rheumatoid Arthritis: ARandomized Controlled Trial. Clin. Ther..

[47] Koivistoinen T., Aatola H., Hutri-Kähönen N., Juonala M., Viikari J.S., Laitinen T., Taittonen L., Lehtimäki T., Kööbi T., Raitakari O.T. (2010). Systemic hemodynamics in young adults with the metabolic syndrome: The Cardiovascular Risk in Young Finns Study. Ann. Med..

[48] Scuteri A., Najjar S.S., Orru M., Usala G., Piras M.G., Ferrucci L., Cao A., Schlessinger D., Uda M., Lakatta E.G. (2010). The central arterial burden of the metabolic syndrome is similar in men and women: The SardiNIA Study. Eur. Heart J..

[49] Lilitkarntakul P., Dhaun N., Melville V., Kerr D., Webb D.J., Goddard J. (2012). Risk factors for metabolic syndrome independently predict arterial stiffness and endothelial dysfunction in patients with chronic kidney disease and minimal comorbidity. Diabetes Care.

[50] Więckowska-Gacek A., Mietelska-Porowska A., Wydrych M., Wojda U. (2021). Western diet as a trigger of Alzheimer’s disease: From metabolic syndrome and systemic inflammation to neuroinflammation and neurodegeneration. Ageing Res. Rev..

[51] Park S.H., Kim J.H., Choi K.H., Jang Y.J., Bae S.S., Choi B.T., Shin H.K. (2013). Hypercholesterolemia accelerates amyloid β-induced cognitive deficits. Int. J. Mol. Med..

[52] Heverin M., Maioli S., Pham T., Mateos L., Camporesi E., Ali Z., Winblad B., Cedazo-Minguez A., Björkhem I. (2015). 27-hydroxycholesterol mediates negative effects of dietary cholesterol on cognition in mice. Behav. Brain Res..

[53] Rutkowsky J.M., Lee L.L., Puchowicz M., Golub M.S., Befroy D.E., Wilson D.W., Anderson S., Cline G., Bini J., Borkowski K. (2018). Reduced cognitive function, increased blood-brain-barrier transport and inflammatory responses, and altered brain metabolites in LDLr -/-and C57BL/6 mice fed a western diet. PLoS ONE.

[54] Wu Y., Chen L., Zhong F., Zhou K., Lu C., Cheng X., Wang S. (2023). Cognitive impairment in patients with heart failure: Molecular mechanism and therapy. Heart Fail. Rev..

[55] Atti A.R., Valente S., Iodice A., Caramella I., Ferrari B., Albert U., Mandelli L., De Ronchi D. (2019). Metabolic Syndrome, Mild Cognitive Impairment, and Dementia: A Meta-Analysis of Longitudinal Studies. Am. J. Geriatr. Psychiatry.

[56] Cerasuolo M., Di Meo I., Auriemma M.C., Trojsi F., Maiorino M.I., Cirillo M., Esposito F., Polito R., Colangelo A.M., Paolisso G. (2023). Iron and Ferroptosis More than a Suspect: Beyond the Most Common Mechanisms of Neurodegeneration for New Therapeutic Approaches to Cognitive Decline and Dementia. Int. J. Mol. Sci..

[57] Andreone B.J., Larhammar M., Lewcock J.W. (2020). Cell Death and Neurodegeneration. Cold Spring Harb. Perspect. Biol..

[58] Silveira Rossi J.L., Barbalho S.M., Reverete De Araujo R., Bechara M.D., Sloan K.P., Sloan L.A. (2022). Metabolic syndrome and cardiovascular diseases: Going beyond traditional risk factors. Diabetes Metab. Res. Rev..

[59] Vozarova B., Weyer C., Lindsay R.S., Pratley R.E., Bogardus C., Tataranni P.A. (2002). High white blood cell count is associated with a worsening of insulin sensitivity and predicts the development of type 2 diabetes. Diabetes.

[60] Akbas E.M., Demirtas L., Ozcicek A., Timuroglu A., Bakirci E.M., Hamur H., Ozcicek F., Turkmen K. (2014). Association of epicardial adipose tissue, neutrophil-to-lymphocyte ratio and platelet-to-lymphocyte ratio with diabetic nephropathy. Int. J. Clin. Exp. Med..

[61] Gijsberts C.M., Ellenbroek G., Ten Berg M.J., Huisman A., Van Solinge W.W., Lam C.S., Asselbergs F.W., Den Ruijter H.M., Pasterkamp G., Hoefer I.E. (2017). Effect of Monocyte-to-Lymphocyte Ratio on Heart Failure Characteristics and Hospitalizations in a Coronary Angiography Cohort. Am. J. Cardiol..

[62] Li L., Ma Y., Geng X.B., Tan Z., Wang J.H., Cui C., Wang H.L., Shang X.M. (2021). Platelet-to-lymphocyte ratio relates to poor prognosis in elderly patients with acute myocardial infarction. Aging Clin. Exp. Res..

